# Neuroendocrine tumors of the gallbladder: a case report and review of the literature

**DOI:** 10.1186/1752-1947-5-334

**Published:** 2011-07-29

**Authors:** Silvia Mezi, Vincenzo Petrozza, Orazio Schillaci, Valentina La Torre, Barbara Cimadon, Martina Leopizzi, Errico Orsi, Filippo La Torre

**Affiliations:** 1 Department of Radiology, Oncology and Human Pathology, Division of Oncology B, "Sapienza" University of Rome, Rome, Italy; 2 Department of Surgical Science and Biotechnology, Division of Pathology, Polo Pontino, "Sapienza" University of Rome, Rome, Italy; 3 Department of Biopathology and Diagnostic Imaging, Division of Nuclear Medicine, University "Tor Vergata", Rome, Italy, and IRCCS NEUROMED, Rome, Italy; 4 Department of Surgical Science, Division of DEA, "Sapienza" University of Rome, Rome, Italy

## Abstract

**Introduction:**

Primary gallbladder neuroendocrine tumors are extremely rare, representing 0.2% of all neuroendocrine tumors. The diagnosis is incidental in most cases.

**Case presentation:**

We describe the case of a 57-year-old Caucasian man who underwent laparoscopic cholecystectomy for the evaluation of a gallbladder polyp that had been incidentally detected by ultasonography. Histologically, his lesion was composed of monomorphic cells that contained small round nuclei and that were organized in small nodular, trabecular, and acinar structures. His cells were positive for chromogranin A and synaptophysin, and a diagnosis of "typical" carcinoid of the gallbladder was made. His post-operative computerized axial tomography, ^111^In-pentetreotide scintigraphy, and hormone-specific marker results were negative. He is disease-free 45 months after surgical treatment.

**Conclusions:**

Characteristic pathological findings of the gallbladder neuroendocrine tumors predict the prognosis. Whereas classical carcinoids of the gallbladder only rarely have a metastatic or invasive phenotype, the "atypical" variants are more aggressive and are associated with a poorer prognosis. Given the difficulty in distinguishing between benign and malignant lesions in the pre-surgical setting, we tend to consider each polypoid-like lesion of the gallbladder to be a high-risk lesion if it is larger than 1 cm and, as a result, to emphasize the need for cholecystectomy in all cases, relying on the pathological and immunohistochemistry analyses for the final diagnosis.

## Introduction

Carcinoids are rare neuroendocrine tumors (NETs) derived from enterochromaffin or Kulchitsky cells, which are widely distributed in the body [1,2]. Consequently, NETs can be found in any location of the body, although the sites most commonly affected are the gastrointestinal and bronchopulmonary tracts, representing approximately 67% and 25% of cases, respectively [3]. NETs are histologically varied entities and can range from indolent, unrecognized neoplasms to highly active, metastatic secretory tumors [4]. Prognostic factors include primary tumor site, histological differentiation, tumor size, angioinvasion, infiltrative growth, and production of hormones [5]. Although the incidence of NETs has increased over the past 30 years, survival has also improved (reviewed by Zuetenhorst and Taal [2]).

According to American epidemiological data, gallbladder (GB) NETs are rare, representing only 0.2% of all NETs [6]. Approximately half of the cases reported in the literature as GB carcinoid tumors appear to be endocrine cell carcinomas, which are histologically and clinically distinct entities [6]. Whereas classical carcinoids of the GB only rarely have a metastatic or invasive phenotype, the "atypical" variants are more aggressive and are associated with a poorer prognosis [6-9]. Here, we describe a case of incidental GB carcinoid tumor in a 57-year-old man.

## Case presentation

**Figure 1:**
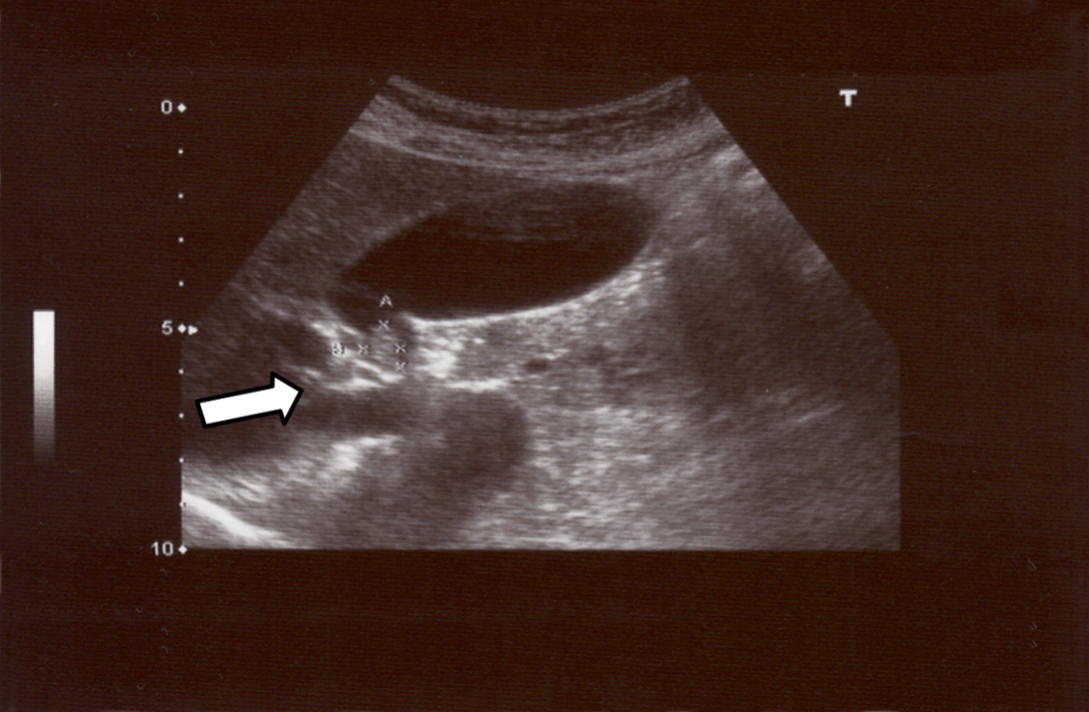
**Abdominal ultrasound image of a polypoid mass between the neck and body of a gallbladder**.

A 57-year-old Caucasian man with a seven-year history of hepatitis B virus infection was admitted to our hospital for the treatment of a GB polyp. The abdominal ultrasonography (US) revealed the presence of a well-defined polypoid mass of approximately 12 × 8 mm in his GB fossa (Figure 1). No evidence of biliary dilatation was noted, and there was no ascites. No image of stones was documented (Figure 1).

On examination, there was no pertinent medical or surgical history, and our patient was asymptomatic and showed no evidence of jaundice. An abdominal examination revealed no tenderness or abnormal mass. The results of laboratory assessments (complete blood count and serum chemistry panel) on admission were normal. On the basis of these data, a pre-operative diagnosis of a single polypoid lesion of the GB (PLG) of larger than 1 cm was made and a laparoscopic cholecystectomy was performed.

**Figure 2:**
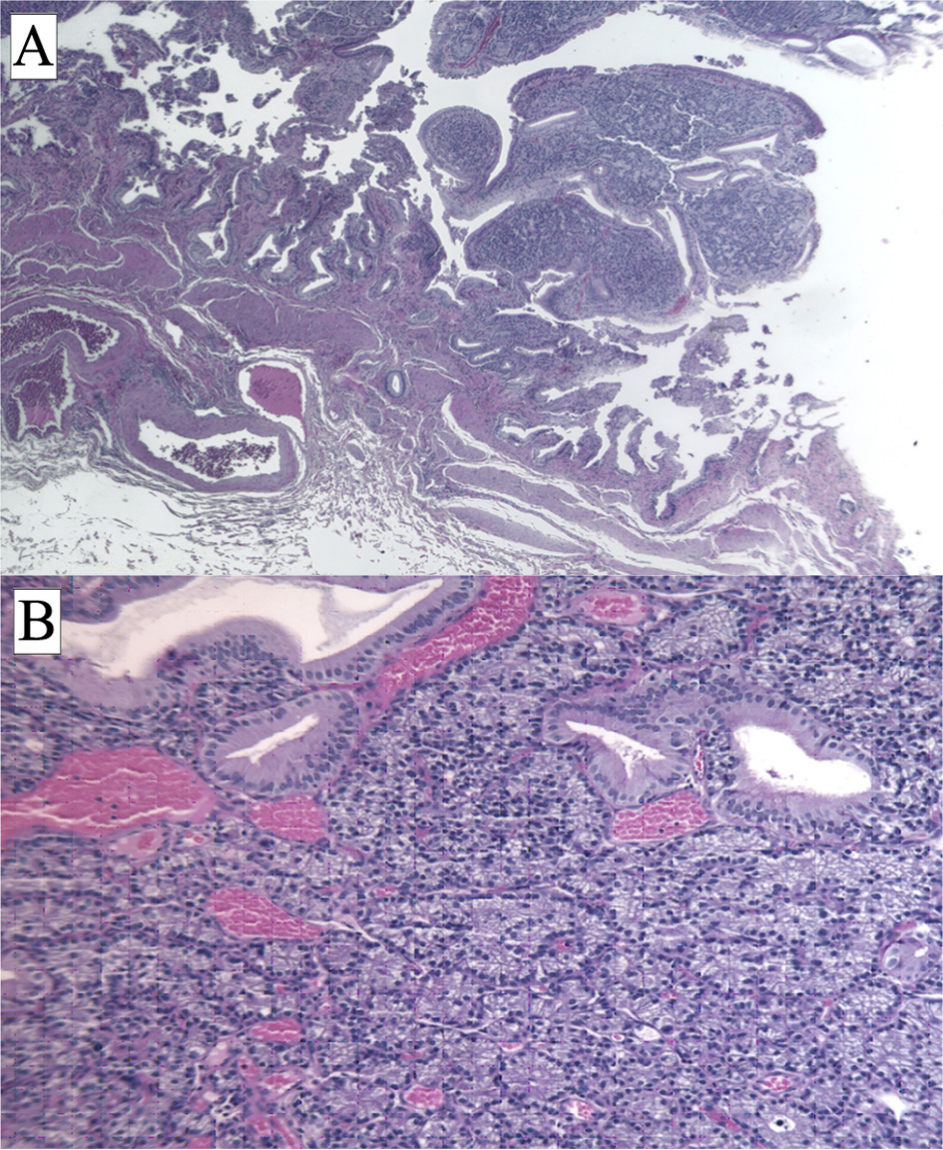
**Hematoxylin-and-eosin section of a carcinoid tumor**. An organ-like growth pattern and rosettes with large cells, prominent nucleoli, and coarse "salt and pepper" chromatin are shown. Magnifications: ×2.5 (A), ×10 (B).

**Figure 3:**
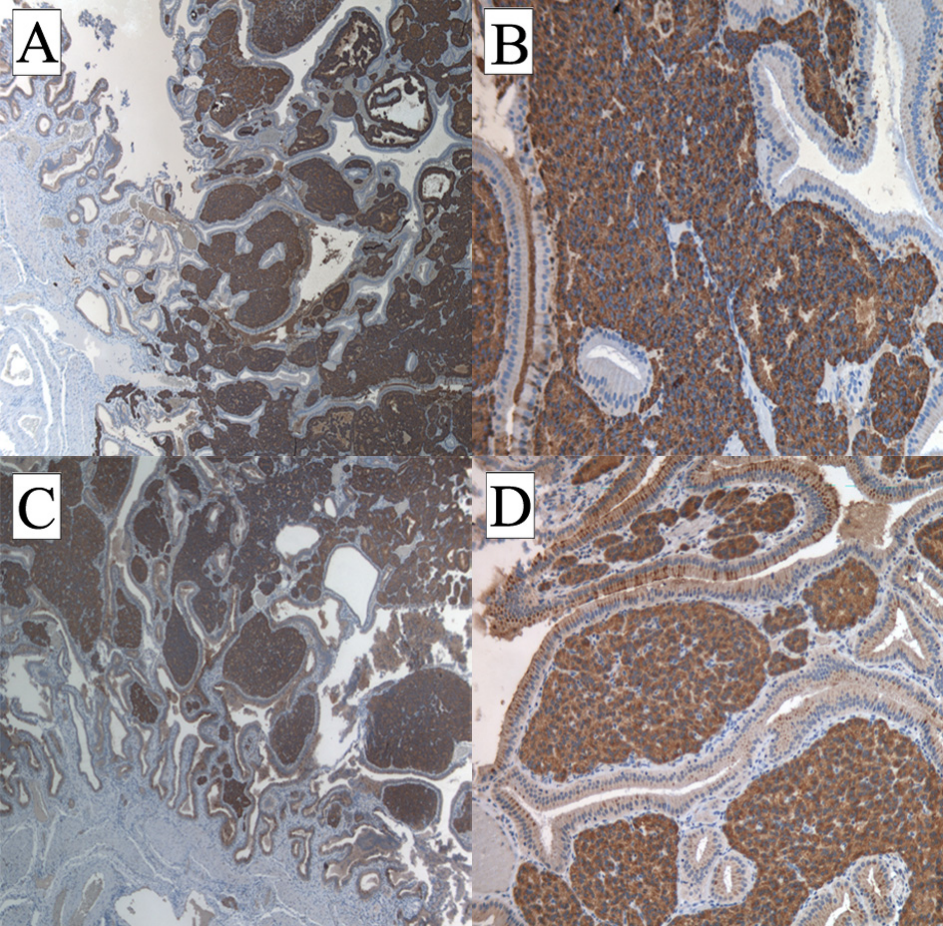
**Tumor cells did not express cytokeratin (A), vimentin (B), CD-31 (C), or CD-34 (D), as revealed by immunohistochemistry**. Endothelial cells positive for CD-31 (C) and CD-34 (D). Magnification: ×20.

**Figure 4:**
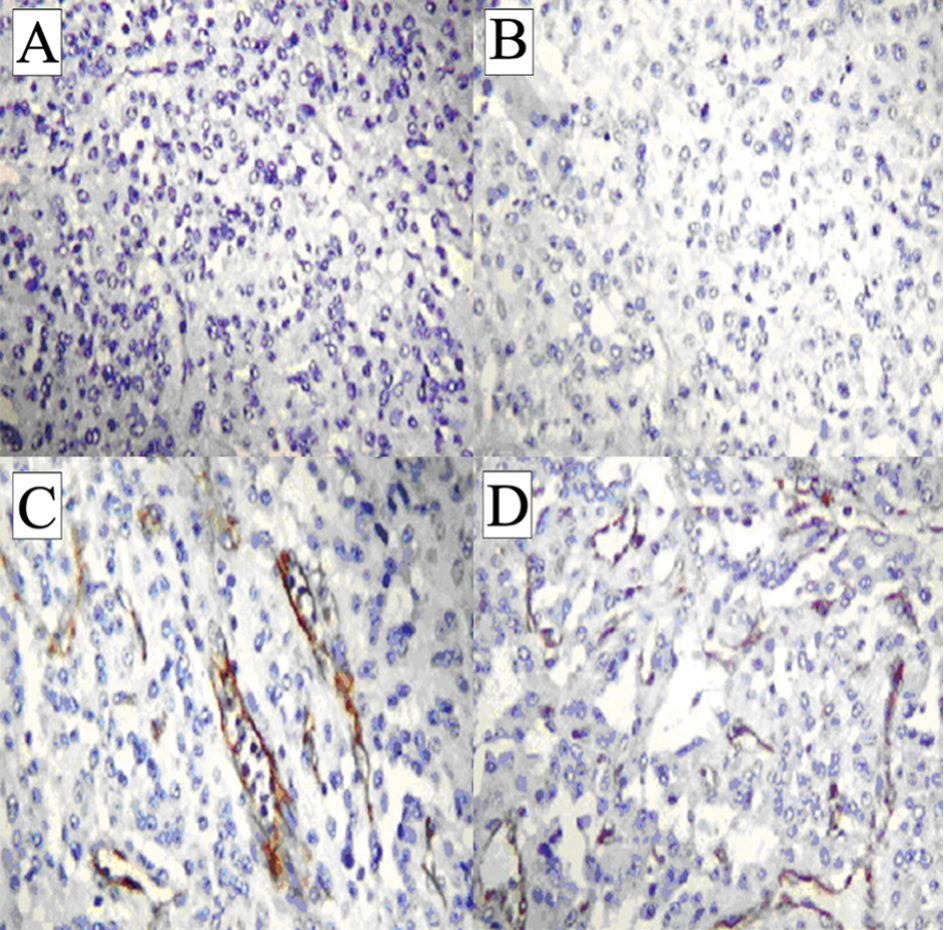
**Tumor cells stained positive for chromogranin A (A, B) and for synaptophysin (C, D)**. Magnifications: ×2.5 (A, C), ×10 (B, D).

On gross inspection, the GB measured 6 cm and no evidence of stones in our patient's lumen was found. However, a polypoid, yellowish lesion, measuring 11 × 8 mm, was found between the body and the neck of his GB. Histologically, his tumor was composed of monomorphic cells containing small, round nuclei and eosinophilic cytoplasm. His cells were organized in small nodular, trabecular, or acinar structures surrounded by a richly vascularized stroma but showed no mitotic structures (Figure 2). Immunohistochemical studies revealed that his cells were negative for cytokeratin, vimentin, and CD-31 and CD-34 (Figure 3). His staining results were positive for tumor cell granules of synaptophysin and chromogranin A (CgA) (Figure 4). Histologically, his GB lesion presented as an NET, and the final diagnosis of "typical carcinoid" was made.

Post-operatively, our patient underwent a total body computed tomography (CT) scan and bone scintigraphy and the results were normal. The results of his ^111^In-pentetreotide scintigraphy, which is used to detect cells with somatostatin receptors, were also normal. His blood levels of glucagon, serotonin, vasoactive intestinal peptide, somatostatin, and gastrin were normal, as were his 24-hour urinary levels of 5-hydroxyindoleacetic acid (5-HIAA) and CgA. After an uneventful recovery, our patient was discharged in good condition, and he is disease-free 45 months after surgical treatment.

## Discussion

Primary GB carcinoids are extremely rare. The first case of a carcinoid tumor of the GB was reported in 1929, and 43 cases of carcinoid tumors have been reported to date. Approximately half of the reported cases of GB carcinoid tumors appear to be endocrine cell carcinomas [3-10]. At present, 278 cases of GB NETs are reported in the Surveillance, Epidemiology, and End Results (SEER) database. Only five well-differentiated NETs are registered in SEER, indicating that the entity of "benign" NETs is very rare in the GB [1]. Neuroendocrine cells derive from local multipotent gastrointestinal stem cells rather than, as initially guessed, by migration by the neural crest. GB NETs may develop from endocrine cells induced by intestinal metaplasia of the body and fundus as well as from pre-existing endocrine cells in the neck of the GB [1-11]. The age at presentation of GB NETs ranges from 38 to 81 years, and there is a markedly higher incidence in women [10]. Carcinoid syndrome is very rare (<1%), and most GB carcinoids are diagnosed incidentally during a histological examination of GB specimens at autopsy, after cholecystectomy for acute or chronic cholecystitis, or after surgery for another suspected biliary pathology [6-8,12-17].

The case reported here was initially diagnosed as a polyp after an ultrasound examination. PLGs are readily detected by US [18] with high specificity (95.8%) [19]. The lifetime prevalence of GB polyps ranges from 1% to 4%. PLGs are "incidentally detected" in approximately 4% to 7% of patients undergoing US of the GB [20], and PLG is one of the most common diseases in biliary surgery.

The majority of GB polyps are non-neoplastic and most commonly include cholesterol polyps (60%) or inflammatory ones (10%). Adenomyomas represent the second most common type of GB polyps (25%). This type of lesion is associated with an increased incidence of GB cancer, and the GB should be removed surgically. Adenonomatous polyps represent a minority. They can progress to cancer, and this risk is related to their size: polyps larger than 1 cm are considered high-risk lesions. The fifth class of GB polyps consists of rare lesions that include heterotopic gastric glands, neurofibromas, carcinoid tumors, leiomyomas, and fibromas.

The specificity of abdominal US in PLG detection is high [19], but the sensitivity of US was reported to be low [21]. Endoscopic US (EUS) may become the standard to define PLGs. Studies have shown a correlation between EUS characteristics and the actual histology of PLGs. EUS is considered to be superior to all types of imaging for GB lesions, particularly for early GB cancer because of the higher operating frequency (7.5 to 12 MHz) that can provide high-resolution images of small lesions and a diagnostic sensitivity for GB malignancy of 90% [21]. High-resolution US (HRUS) has demonstrated a diagnostic sensitivity of as high as 90% and an accuracy of 62.9% for staging the depth of cancer invasion [22]. Both EUS and HRUS minimize the changes of not identifying pre-malignant lesion. If the polyps are severe or appear malignant or if large or irregular lesions are found, a CT scan should be performed in order to avoid missing a GB carcinoma. Pre-operative suspicion and a differential diagnosis of GB cancer are very important for selecting the optimal treatment. CT could be used not only to distinguish an early GB carcinoma from a PLG but also to assess the tissue around the malignant PLG and regional lymph node metastases [19]. Although imaging such as US, EUS, or CT has been widely used, it is still difficult to differentiate cancer from non-neoplastic lesions before an operation. Hence, differentiating a pre-cancerous lesion from early GB cancer is essential. The risk of malignancy is between 45% and 67% in polyps from 1 to 1.5 cm in size [21].

Operative indications for PLGs included a maximal diameter of 1 cm, a wide-base lesion, lesions tending to become enlarged in a short period, patient age of more than 50 years, a single polypoid lesion, coexisting GB stones, and a PLG associated with irregular thickening of the local GB wall.

Our patient's histological results after cholecystectomy were suggestive of an NET tumor. The determination of the histological type of the tumor and differential diagnosis from GB adenocarcinoma are often difficult. The identification of neuroendocrine cells and the immunohistochemical expression of marker proteins as well as other cell type-specific amines and peptides are necessary to define a GB NET. Our patient's immunohistochemistry test results were negative for cytokeratin, vimentin, and CD-31 and CD-34, allowing us to exclude a likely diagnosis of adenocarcinoma, sarcoma, or vascular tumor, respectively. The combination of the high histological differentiation, the tumor size, the absence of angioinvasion and infiltrative growth, and the immunohistochemical staining supported the final diagnosis of a "typical" rather than of an "atypical" NET tumor.

When feasible, surgical treatment, with the goal of complete resection, is the gold standard for typical carcinoids of the GB. For pre-invasive and early-detected cancer (T1s and T1), simple cholecystectomy is probably an adequate therapy. For advanced lesions, a more aggressive radical surgery, including radical cholecystectomy and regional lymphadenectomy combined with a hepatic resection in order to obtain adequate free margins, is needed [1]. Additional therapies in an adjuvant setting are not required for typical carcinoids according to the low metastatic potential of the neoplasia as well as to the general insensitivity to traditional radiotherapy and chemotherapy in low-grade cancer disease.

For many years, sieric CgA and urinary 5-HIAA, each of which has a specificity of nearly 100% but a low sensitivity, have been the gold standard for detecting carcinoids and conducting follow-up [23]. ^111^In-pentetreotide has a high affinity for somatostatin subtype 2 and 5 receptors, which are present on the cell membranes of carcinoid tumor cells, making ^111^In-pentetreotide scintigraphy a good technique for imaging carcinoid tumors [24]. Standard bone scintigraphy has a higher sensitivity for the detection of bone metastases in patients with carcinoid tumors [25]. Post-operative specific tumor markers, total body CT, ^111^In-pentetreotide scintigraphy, and bone scintigraphy tests in our patient were all normal, indicating the lack of metastases and the successful surgical treatment of a "typical" carcinoid of the GB. Indeed, in one study, 82.4% of GB carcinoids remained localized and only 11.8% of patients demonstrated distant metastases [3]. The same source reported a five-year survival of 60.8% ± 14.8%. Modlin and colleagues [1] reported a median survival of 9.8 months among 278 cases of GB NETs reported in SEER. The five-year survival rates for tumors classified as carcinoids-neuroendocrine carcinoma or small-cell cancer were 36.9% and 0%, respectively [1].

## Conclusions

Considering the difficulties in making a pre-operative differential diagnosis between a benign "typical" carcinoid and the more aggressive "atypical" variants or between NET, adenocarcinoma, and benign lesions of the GB, we emphasize the need for surgical management for any suspected polypoid lesion, relying on the pathologist and immunohistochemistry analyses for the final diagnosis. We underline the need to distinguish between different forms of NETs of the GB with different metastatic potential, prognosis, and clinical course.

## Abbreviations

5-HIAA: 5-hydroxyindoleacetic acid; CgA: chromogranin A; CT: computed tomography; EUS: endoscopic ultrasonography; GB: gallbladder; HRUS: high-resolution ultrasonography; NET: neuroendocrine tumor; PLG: polypoid lesion of the gallbladder; SEER: Surveillance, Epidemiology, and End Results; US: ultrasonography.

## Consent

Written informed consent was obtained from the patient for publication of this case report and any accompanying images. A copy of the written consent is available for review by the Editor-in-Chief of this journal.

## Competing interests

The authors declare that they have no competing interests.

## Authors' contributions

SM drafted and wrote the manuscript and was involved in data interpretation. EO was involved in the conception and design of the study. FLT and VLT were involved in the care of our patient. VP was involved in histological diagnosis, pathological findings, immunohistochemical studies, and figures and contributed to writing the manuscript according to his specialty. ML was involved in immunohistochemical studies and figures. OS provided scintigraphic images and was responsible for critical revision of CT images. BC was involved in administrative support. All authors read and approved the final manuscript.
